# Sedentary time associates detrimentally and physical activity beneficially with metabolic flexibility in adults with metabolic syndrome

**DOI:** 10.1152/ajpendo.00338.2023

**Published:** 2024-02-28

**Authors:** Taru Garthwaite, Tanja Sjöros, Saara Laine, Mikko Koivumäki, Henri Vähä-Ypyä, Tiina Verho, Jooa Norha, Petri Kallio, Maria Saarenhovi, Eliisa Löyttyniemi, Harri Sievänen, Noora Houttu, Kirsi Laitinen, Kari K. Kalliokoski, Tommi Vasankari, Juhani Knuuti, Ilkka Heinonen

**Affiliations:** ^1^Turku PET Centre, University of Turku, Åbo Akademi University, and Turku University Hospital, Turku, Finland; ^2^The UKK Institute for Health Promotion Research, Tampere, Finland; ^3^Department of Clinical Physiology and Nuclear Medicine, University of Turku and Turku University Hospital, Turku, Finland; ^4^Paavo Nurmi Centre and Unit for Health and Physical Activity, University of Turku, Turku, Finland; ^5^Department of Biostatistics, University of Turku and Turku University Hospital, Turku, Finland; ^6^Institute of Biomedicine, University of Turku, Turku, Finland; ^7^Faculty of Medicine and Health Technology, Tampere University, Tampere, Finland

**Keywords:** energy metabolism, metabolic flexibility, metabolic syndrome, physical activity, sedentary behavior

## Abstract

Metabolic flexibility (MetFlex) describes the ability to respond and adapt to changes in metabolic demand and substrate availability. The relationship between physical (in)activity and MetFlex is unclear. This study aimed to determine whether sedentary time, physical activity (PA), and cardiorespiratory fitness associate with MetFlex. Sedentary time, standing, and PA were measured with accelerometers for 4 weeks in 64 sedentary adults with metabolic syndrome [37 women, 27 men; 58.3 (SD 6.8) years]. Fitness (V̇o_2max_; mL·kg^−1^·min^−1^) was measured with graded maximal cycle ergometry. MetFlex was assessed with indirect calorimetry as the change in respiratory exchange ratio (ΔRER) from fasting to insulin stimulation with hyperinsulinemic-euglycemic clamp and from low-intensity to maximal exercise. Carbohydrate (CHOox) and fat oxidation (FATox) were calculated from respiratory gases. High sedentary time associated with higher fasting RER [β = 0.35 (95% confidence interval: 0.04, 0.67)], impaired insulin-stimulated MetFlex (ΔRER) [β=−0.41 (−0.72, −0.09)], and lower fasting FATox [β=−0.36 (−0.67, −0.04)]. Standing associated with lower fasting RER [β=−0.32 (−0.62, −0.02)]. Higher standing time and steps/day associated with higher fasting FATox [β = 0.31 (0.01, 0.61), and β = 0.26 (0.00, 0.53)]. Light-intensity and total PA associated with better insulin-stimulated MetFlex [β = 0.33 (0.05, 0.61)], and β = 0.33 (0.05, 0.60)]. Higher V̇o_2max_ associated with higher CHOox during maximal exercise [β = 0.81 (0.62, 1.00)], as well as during insulin stimulation [β = 0.43 (0.13, 0.73)]. *P* values are less than 0.05 for all associations. Sedentary time and PA associate with MetFlex. Reducing sitting and increasing PA of even light intensity might aid in the prevention of metabolic diseases in risk populations through their potential effects on energy metabolism.

**NEW & NOTEWORTHY** High accelerometer-assessed sedentary time associates with metabolic inflexibility measured during hyperinsulinemic-euglycemic clamp in adults with metabolic syndrome, and more light-intensity and total physical activity associate with more metabolic flexibility. Physical activity behaviors may thus play an important role in the regulation of fuel metabolism. This highlights the potential of reduced sedentary time and increased physical activity of any intensity to induce metabolic health benefits and help in disease prevention in risk populations.

## Graphical abstract

**Figure:**
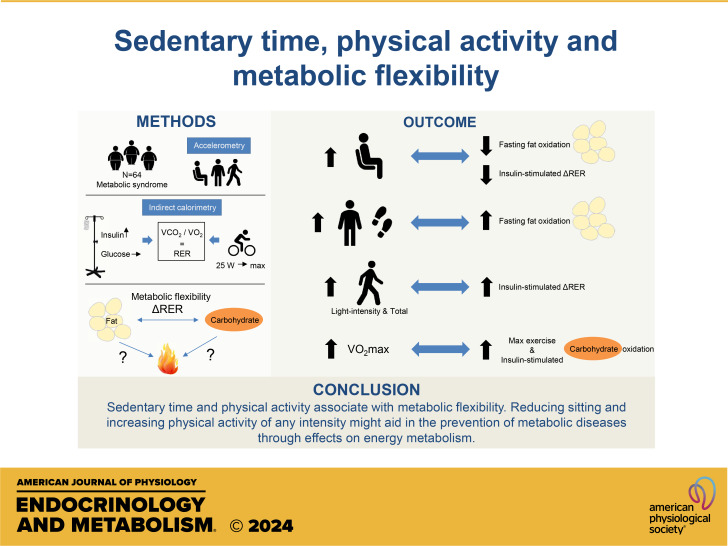

## INTRODUCTION

Metabolic flexibility (MetFlex) describes the ability to respond and adapt to changes in metabolic demand and substrate availability (1). MetFlex is needed to maintain a constant and sufficient energy supply to tissues and cells, and it plays an important role in overall metabolic health (1). Impairments in fuel oxidation and storage, as well as in the ability to switch between the main energy substrates, carbohydrates (CHO), and fats, have been associated with obesity, insulin resistance, metabolic syndrome, and type 2 diabetes (2–4).

MetFlex was originally assessed with the arteriovenous leg balance technique during insulin stimulation by hyperinsulinemic-euglycemic clamp (HEC) and expressed as the change in respiratory quotient (ΔRQ = insulin-stimulated RQ − fasting RQ) (2). RQ thus represents substrate metabolism at the tissue level. Therefore, the respiratory exchange ratio (RER) determined from respiratory gases collected with less invasive indirect calorimetry is often used interchangeably with RQ to represent whole body fuel metabolism, as RER = RQ over time. Fasting RQ, or RER, itself is also often used as a marker of MetFlex, and more recently alternative indicators have been proposed as well, e.g., variability in RQ and insulin after a meal (5). RQ represents the relative contribution of CHO and fat to total fuel oxidation, with low values (i.e., close to 0.7) indicating high fat oxidation (FATox) and high values (i.e., close to 1.0) indicating higher CHO oxidation (CHOox) or impaired FATox (6). In healthy individuals, FATox is greater during fasting, whereas after a meal or in response to insulin stimulation, FATox is suppressed, and CHO uptake, oxidation, and storage increase (2). In contrast, in obesity-induced insulin resistance, FATox at a fasting state is lower, and the ability to switch to CHOox in response to insulin stimulation is impaired. This inability to adapt fuel oxidation to fuel availability has been defined as metabolic inflexibility (2). A few suggested contributing factors are muscle lipid accumulation and impairments in glucose disposal rate, adipose tissue lipid flux, and mitochondrial oxidative capacity, but the causal links are yet to be established (7).

Lifestyle factors have been suggested to play an important role in the development of these metabolic impairments, and some studies indicate improvements in MetFlex following lifestyle changes such as aerobic exercise training and weight loss (8, 9). On the other hand, physical inactivity and sedentary time have been proposed as primary determinants of metabolic inflexibility (5, 10, 11). However, the current evidence regarding the role of sedentary time in the regulation of MetFlex is mainly based on experimental studies that have used bed rest models to simulate inactivity and sedentary time and assessed MetFlex with surrogate or alternative measures (5, 10, 11). Studies directly examining the role of device-measured physical activity (PA) and sedentary time in free-living conditions in the regulation of MetFlex, as measured by indirect calorimetry during HEC, are lacking.

Sedentary time is detrimentally associated with several metabolic health markers, including glucose and lipid outcomes (12, 13); thus it is important to understand the effects of sedentariness on the regulation of fat and CHO oxidation under varying physiological conditions. Although the causality is unclear, impairments in fuel metabolism and lipid accumulation might be the primary underlying factors preceding obesity and insulin resistance. Consequently, studying the role of sedentary time and physical (in)activity in energy metabolism is particularly important in populations already at an increased risk of metabolic diseases. Therefore, the primary objective of this study was to investigate the associations of accelerometer-measured sedentary time, PA, and cardiorespiratory fitness with MetFlex measured by indirect calorimetry during HEC in physically inactive sedentary adults with metabolic syndrome.

## MATERIALS AND METHODS

### Study Design

The data in this cross-sectional study is a part of the baseline data of an intervention study (Clinicaltrials.gov NCT03101228). The data were collected at the Turku PET Center (Turku, Finland) between 2017 and 2019. The study was approved by the Ethics Committee of the Hospital District of Southwest Finland (16/1801/2017), and good clinical practice and the Declaration of Helsinki were followed. A written informed consent was obtained from all participants before entering the study.

### Participants

As previously reported (14), participants were recruited from the local community by newspaper advertisements and bulletin leaflets. The inclusion criteria were age 40–65 yr, physical inactivity [<120 min/week of self-reported moderate-to-vigorous PA (MVPA)], sedentary time ≥10 h/day or ≥60% of accelerometer wear time/day during screening, body mass index (BMI) 25–40 kg·m^−2^, blood pressure <160/100 mmHg, fasting glucose <7.0 mmol·L^−1^, and fulfillment of metabolic syndrome criteria (15) including at least three of the following: waist circumference ≥94 cm for men/≥80 cm for women, triglycerides ≥1.7 mmol·L^−1^, HDL <1.0 mmol·L^−1^ for men/<1.3 mmol·L^−1^ for women, systolic blood pressure ≥130 and/or diastolic blood pressure ≥85 mmHg, or fasting glucose ≥5.6 mmol·L^−1^. The exclusion criteria were a previous cardiac event; diagnosed diabetes; abundant alcohol consumption (according to national guidelines); use of narcotics, cigarette or snuff tobacco; depressive or bipolar disorder; and any chronic disease or condition that could endanger participant safety or study procedures, or interfere with the interpretation of results. Sixty-four participants were recruited according to the sample size calculation for the whole intervention study (14).

### Accelerometry

Tri-axial accelerometers (UKK AM30, UKK Terveyspalvelut Oy, Tampere, Finland) were worn on the right hip during waking hours (except when exposed to water) for 4 consecutive weeks to assess sedentary time, breaks in sedentary time, standing, and PA. Wear time of 10–19 h/day and at least 4 days of measurement were considered valid. The accelerometer data were analyzed in 6-second epochs with validated mean amplitude deviation and angle for posture estimation methods as described previously (12, 16, 17). Sedentary time and standing were defined as ≤1.5 metabolic equivalents (METs) and identified and differentiated with the angle for the posture estimation method. Light-intensity PA (LPA) was defined as 1.5–2.9 METs and MVPA as ≥3.0 METs.

### Cardiorespiratory Fitness

Cardiorespiratory fitness was assessed with a graded maximal cycle ergometry test (eBike EL Ergometer + CASE v6.7, GE Medical Systems Information Technologies Inc., Milwaukee, WI) with direct respiratory gas measurements (Vyntus CPX, CareFusion, Yorba Linda, CA). The test protocol and V̇o_2max_ determination criteria have been described in detail previously (18). In short, the cycling pace was ∼65 rpm, and the intensity started at 25 W and was increased by 25 W every three minutes until volitional exhaustion. V̇o_2max_ (mL·kg^−1^·min^−1^), V̇o_2max_ per fat-free mass (mL·kg_FFM_^−1^·min^−1^), and maximal power output (PO; W) were determined as measures of cardiorespiratory fitness. Complete fitness data are available for 58 participants, as the test was stopped early for five participants before reaching volitional exhaustion (due to knee pain, hip pain, abnormal increase in blood pressure, or dyspnea), and the results of one participant were lost due to technical issues.

### Hyperinsulinemic-Euglycemic Clamp

HEC was performed after an overnight fast as previously described (14). Plasma insulin was increased above normal physiological level by insulin (100 U·mL^−1^ Actrapid; Novo Nordisk, Bagsvaerd, Denmark) administration at a steady 40 mU·min^−1^·m^−2^ body surface area rate after priming with higher doses. A 20% glucose infusion was started 4 minutes after starting the insulin infusion. Blood samples were collected every 5–10 min to measure plasma glucose concentration (Analox GM7, Analox Instruments, London, UK), and the infusion rate was adjusted accordingly to maintain an ∼5.0 mmol·L^−1^ concentration. Whole body glucose uptake (M value; mg·kg^−1^·min^−1^) was calculated in 20-min intervals from steady-state glucose values.

### Indirect Calorimetry

Indirect calorimetry was performed with a ventilated hood system (Quark RMR + OMNIA, COSMED, Rome, Italy) at rest after an overnight fast and during the insulin stimulation by HEC. Participants were instructed to avoid strenuous physical exertion, caffeine, and alcohol for 24 h before the research visit and to minimize PA on the morning of measurement by arriving at the research facility by car or by bus. Before the measurement, participants rested in a lying position for a minimum of 20 min. Respiratory gas exchange was measured breath-by-breath with an automated respiratory gas analyzer for 20 (SD 2) min in the fasting state, and for 14 (SD 2) min during HEC, starting at 29 (SD 8) min after the initiation of HEC. The first 4 minutes were discarded, and steady-state was determined by <10% coefficient of variation (CV) in oxygen consumption (V̇o_2_) and carbon dioxide production (V̇co_2_) and/or <5% CV in RER (=V̇co_2_/V̇o_2_) for ≥4 min. ΔRER from fasting to insulin stimulation (=insulin-stimulated RER – fasting RER) was calculated from averaged steady-state RER values and defined as the measure of MetFlex. CHO and fat oxidation (mg·kg^−1^·min^−1^, and %energy expenditure (EE)] were calculated from V̇o_2_ and V̇co_2_, with the assumption of negligible protein oxidation (19):

$$\text{CHOox }(\text{g}\cdot {\text{min}}^{-1})=4.55\times \overset{\cdot }{\text{V}}{\text{CO}}_{2}(\text{L}\cdot {\text{min}}^{-1})-3.21\times \overset{\cdot }{\text{V}}o_{2}(\text{L}\cdot {\text{min}}^{-1})$$

$$\text{FATox }(\text{g}\cdot {\text{min}}^{-1})=1.67\times \overset{\cdot }{\text{V}}{\text{O}}_{2}(\text{L}\cdot {\text{min}}^{-1})-1.67\times \overset{\cdot }{\text{V}}{\text{CO}}_{2}(\text{L}\cdot {\text{min}}^{-1})$$

The CHOox rate was subtracted from the glucose infusion rate to estimate non-oxidative glucose disposal. Negative values were interpreted as zero. CHOox exceeding the amount of exogenous glucose was assumed to represent the oxidation of other CHO sources, i.e., endogenous glucose, glycogen, and lactate.

Fasting calorimetry data from two participants were excluded from analyses because one participant did not reach a steady state, and the results of one participant were unreliable due to anxiety during calorimetry, a consequent premature end to the measurement, and incomplete data.

MetFlex was also estimated during exercise from respiratory gases collected breath-by-breath with a mask (Vyntus CPX, CareFusion, Yorba Linda, CA) continuously throughout the maximal cycle ergometer test described above. V̇o_2_, V̇co_2_, and RER values were averaged over 20-s periods, and the minimum and maximum RER during the test were used as outcome measures and used to calculate the ΔRER from the low-intensity (25 W) to maximal exercise. To calculate EE and substrate oxidation rates, the following formulas were used, accounting for the shift in acid-base balance and an increased contribution of muscle glycogen to CHOox at higher exercise intensities (20):

Low-intensity exercise:

$$\text{EE }(\text{kcal}\cdot {\text{min}}^{-1})=0.575\times \overset{\cdot }{\text{V}}{\text{CO}}_{2}(\text{L}\cdot {\text{min}}^{-1})+4.435\times \overset{\cdot }{\text{V}}{\text{O}}_{2}(\text{L}\cdot {\text{min}}^{-1})$$

$$\text{CHOox }(\text{g}\cdot {\text{min}}^{-1})=4.344\times \overset{\cdot }{\text{V}}{\text{CO}}_{2}(\text{L}\cdot {\text{min}}^{-1})-3.061\times \overset{\cdot }{\text{V}}{\text{O}}_{2}(\text{L}\cdot {\text{min}}^{-1})$$

Maximal exercise:

$$\text{EE }(\text{kcal}\cdot {\text{min}}^{-1})=0.550\times \overset{\cdot }{\text{V}}{\text{CO}}_{2}(\text{L}\cdot {\text{min}}^{-1})+4.471\times \overset{\cdot }{\text{V}}{\text{O}}_{2}(\text{L}\cdot {\text{min}}^{-1})$$

$$\text{CHOox }(\text{g}\cdot {\text{min}}^{-1})=4.210\times \overset{\cdot }{\text{V}}{\text{CO}}_{2}(\text{L}\cdot {\text{min}}^{-1})-2.962\times \overset{\cdot }{\text{V}}{\text{O}}_{2}(\text{L}\cdot {\text{min}}^{-1})$$

FATox (g·min^−1^) was calculated as 1.695 × V̇o_2_ (L·min^−1^) – 1.701 × V̇co_2_ (L·min^−1^) at both intensities, and all calculations assumed negligible protein oxidation. Delta exercise efficiency was also calculated from exercise PO and EE (kcal·min^−1^) as ΔPO/ΔEE × 100 (21). PO was converted from watts to kcal/min using a conversion factor of 0.014. For women, the efficiency was calculated between 25 and 75 W and for men between 25 and 100 W to represent efficiency at moderate-intensity exercise, as the chosen upper limits correspond to 65% and 66% of mean maximal PO, respectively.

### Dietary Intake

The intakes of energy, CHO, fat, and protein were assessed with 4-day food diaries (including one weekend day). The daily means for total energy and macronutrient intake were calculated by software (AivoDiet 2.2.0.1, Aivo, Turku, Finland) utilizing the food composition database provided by the Finnish National Institute for Health and Welfare (www.fineli.fi/fineli/en).

### Metabolic and Anthropometric Outcomes

Venous blood samples were drawn after fasting for at least 10 h and analyzed at the Turku University Hospital Laboratory. Plasma insulin was measured by electrochemiluminescence immunoassay (Cobas 8000 e801), plasma glucose by enzymatic reference method with hexokinase GLUC3, and cholesterol (total, LDL, and HDL), triglycerides, free fatty acids and lactate by enzymatic colorimetric tests (Cobas 8000 c702); all analyzers by Roche Diagnostics, Mannheim, Germany. Homeostatic model assessment of insulin resistance (HOMA-IR) index was calculated with the following formula: fasting glucose (mmol·L^−1^) × fasting insulin (mU·L^−1^)/22.5. Blood samples were also collected during HEC (∼80 min from the start) to determine the levels of free fatty acids and lactate and their insulin-stimulated changes from baseline.

Body weight was measured, and body fat percent and fat-free mass were estimated by air displacement plethysmography (Bod Pod, COSMED USA Inc., Concord. CA) after at least a 4-h fast. Height was measured with a stadiometer, and BMI was calculated from weight and height (kg·m^−2^). Waist circumference was measured midway between the iliac crest and the lowest rib.

### Statistics

Descriptive statistics including means (SD), or for nonnormally distributed outcomes medians with lower and upper quartiles (Q1, Q3), were calculated, and the differences between sexes were tested with unpaired *t* test. Correlations of MetFlex and substrate oxidation variables with metabolic, anthropometric, and dietary outcomes were evaluated with Pearson partial correlation analysis adjusting for sex and age. The changes in RER and substrate oxidation from fasting to insulin stimulation and from low-intensity to maximal exercise were assessed with paired *t* test. Insulin-stimulated changes in blood sample-derived metabolic outcomes were also assessed. Unpaired *t* test was used to compare ΔRER between two evenly divided groups according to daily sedentary time (≤10.0 h/day vs. >10.0 h/day; *n* = 32 in both). The associations of sedentary time, PA, and cardiorespiratory fitness with MetFlex and substrate oxidation were examined with multivariable regression models. The model always included one MetFlex or substrate oxidation variable as the dependent variable, and the accelerometer or fitness outcomes were entered into the model one at a time as the independent variable. The regression model was adjusted for sex and age, and all models with accelerometry outcomes were adjusted for accelerometer wear time. The models examining the associations of sedentary time, standing, and steps with MetFlex and substrate oxidation were further adjusted for total PA. The regression results are expressed as standardized β-coefficients (95% confidence interval). The normal distribution of the residuals was evaluated visually, and log10 transformations were performed as needed. Variance inflation factors <5 were considered not to have multicollinearity issues. Statistical significance was set at *P* < 0.05 (two-tailed). Analyses were performed with IBM SPSS Statistics 27.0 (IBM Corp., Armonk, NY) and JMP Pro 16.0.0 (SAS Institute Inc., Cary, NC). The figures were created with JMP Pro 16.0.0 and GraphPad Prism 5.01 (GraphPad Software, San Diego, CA).

## RESULTS

The descriptive and metabolic characteristics of participants (*n* = 64; 37 women, 27 men) are presented in Tables 1–3. The mean age of participants was 58.3 (SD 6.8) years, and the mean BMI was 31.6 (SD 4.3) kg·m^−2^. The participants spent on average 10.0 (SD 1.0) h/day sedentary, 1.8 (SD 0.6) h/day standing, and 2.7 (SD 0.6) h/day in PA, taking 5,149 (SD 1,825) steps/day (Table 1).

**Table 1.** Descriptive characteristics of the participants

	Total	Men	Women
*n*, %total	64 (100)	27 (42)	37 (58)
Age, years	58.3 (6.8)	58.6 (6.0)	58.0 (7.4)
Weight, kg	93.2 (16.1)	101.2 (16.5)	87.4 (13.1)***
BMI, kg·m^−2^	31.6 (4.3)	31.6 (4.5)	31.6 (4.2)
Waist circumference, cm	110.9 (11.3)	115.5 (12.5)	107.5 (9.0)**
Body fat, %	43.1 (7.9)	37.2 (7.6)	47.4 (4.7)***
Systolic blood pressure, mmHg	143 (16)	141 (16)	144 (16)
Diastolic blood pressure, mmHg	88 (8)	88 (10)	88 (7)
Activity and fitness outcomes			
Accelerometry, days	26 (4)	25 (4)	26 (3)
Wear time, h/day	14.54 (0.97)	14.33 (1.06)	14.69 (0.89)
Sedentary time, h/day	10.04 (1.01)	10.20 (1.08)	9.93 (0.95)
Standing, h/day	1.79 (0.59)	1.47 (0.44)	2.02 (0.58)***
LPA, h/day	1.74 (0.44)	1.63 (0.50)	1.82 (0.38)
MVPA, h/day	0.97 (0.32)	1.03 (0.39)	0.92 (0.26)
Total PA, h/day	2.70 (0.62)	2.66 (0.74)	2.74 (0.53)
Steps/day	5,149 (1,825)	5,329 (2,084)	5,018 (1,629)
Breaks in sedentary time/day	29 (8)	26 (7)	30 (9)*
V̇o_2max_,^a^ mL·kg^−1^·min^−1^	22.7 (4.7)	25.0 (4.9)	21.1 (3.7)**
V̇o_2max_,^a^ mL·kg_FFM_^−1^·min^−1^	40.0 (6.1)	39.9 (6.2)	40.0 (6.1)
Dietary intake			
Energy intake, kcal·day^−1^	1,797 (399)	1,910 (444)	1,715 (346)
CHO, %energy intake/day	39.2 (7.6)	38.7 (7.4)	39.5 (7.8)
Fat, %energy intake/day	38.8 (6.5)	38.4 (6.1)	39.1 (6.8)
Protein, %energy intake/day	17.8 (2.8)	18.1 (3.1)	17.6 (2.7)

Values are means (SD). BMI, body mass index; LPA, light-intensity physical activity; MVPA, moderate-to-vigorous physical activity; PA, physical activity; V̇o_2max_, maximal oxygen consumption; FFM, fat-free mass; CHO, carbohydrate; *P < 0.05, **P < 0.01, ***P < 0.001 between sexes. ^a^Data available for 58 participants.

The median fasting RER was 0.91 (Q1 0.85, Q3 0.98), and the mean insulin-stimulated RER was 0.92 (SD 0.10) (Table 2). The average difference between fasting and insulin stimulation was nonsignificant (*P* = 0.38). CHOox increased slightly, but nonsignificantly, from fasting to insulin stimulation [2.5 (SD 0.9) to 2.6 (1.1) mg·kg^−1^·min^−1^], with no change in FATox (*P* > 0.05 for both) (Table 2). CHOox represented the majority of insulin-stimulated glucose disposal, as the calculated estimate of mean nonoxidative glucose disposal was 0.6 (SD 1.3) mg·kg^−1^·min^−1^. The total CHOox was calculated to consist of 76% exogenous glucose disposal and 24% from other CHO sources. During HEC, free fatty acids decreased by −0.48 (SD 0.20) mmol·L^−1^, and lactate increased by 0.18 (0.30) mmol·L^−1^, on average (*P* < 0.001 for both).

**Table 2.** Metabolic characteristics of the participants during fasting and insulin stimulation

	Total	Men	Women
Insulin, pmol·L^−1^			
Fasting^a^	69.5 (48.6, 104.2)	90.3 (55.6, 173.6)	55.6 (48.6, 83.3)###
HEC	503.7 (100.9)***	528.8 (110.8)	484.4 (89.4)
Glucose, mmol·L^−1^			
Fasting	5.9 (0.4)	6.0 (0.5)	5.7 (0.3)#
HEC	5.3 (0.3)***	5.4 (0.4)	5.2 (0.3)##
Free fatty acids, mmol·L^−1^			
Fasting	0.60 (0.20)	0.53 (0.18)	0.66 (0.20)#
HEC	0.12 (0.06, 0.18)***	0.14 (0.09, 0.20)	0.09 (0.05, 0.16)
Lactate, mmol·L^−1^			
Fasting	1.0 (0.8, 1.3)	1.0 (0.9, 1.2)	1.0 (0.8, 1.3)
HEC	1.2 (1.1, 1.4)***	1.1 (1.0, 1.2)	1.3 (1.1, 1.5)#
V̇o_2,_ mL·min^−1^			
Fasting^b^	240 (44)	272 (43)	219 (29)###
HEC	253 (44)***	282 (45)	233 (31)###
V̇co_2_, mL·min^−1^			
Fasting^b^	215 (198, 240)	242 (212, 262)	203 (187, 216)###
HEC	224 (204, 253)***	253 (219, 279)	213 (198, 233)###
RER			
Fasting^b^	0.91 (0.85, 0.98)	0.87 (0.83, 0.97)	0.93 (0.86, 0.99)
HEC	0.92 (0.10)	0.90 (0.10)	0.93 (0.09)
ΔRER (HEC − fasting)^b^	0.00 (−0.04, 0.03)	−0.01 (−0.03, 0.01)	0.00 (−0.05, 0.05)
EE, kcal·day^−1^			
Fasting^b^	1,697 (297)	1,912 (298)	1,552 (191)###
HEC	1,788 (300)***	1,982 (304)	1,647 (206)###
CHO oxidation, mg·kg^−1^·min^−1^			
Fasting^b^	2.5 (0.9)	2.4 (0.9)	2.6 (0.9)
HEC	2.6 (1.1)	2.4 (1.2)	2.7 (1.1)
CHO, %EE			
Fasting^b^	69.1 (47.8, 91.9)	55.4 (41.1, 89.6)	76.2 (51.7, 92.7)
HEC	68.1 (48.1, 91.4)	53.4 (45.1, 91.1)	72.4 (57.3, 92.0)
Fat oxidation, mg·kg^−1^·min^−1^			
Fasting^b^	0.4 (0.4)	0.4 (0.4)	0.3 (0.4)
HEC	0.4 (0.4)	0.5 (0.5)	0.3 (0.4)
Fat, %EE			
Fasting^b^	30.9 (8.1, 52.2)	44.6 (10.4, 58.9)	23.8 (7.3, 48.3)
HEC	31.9 (8.6, 51.9)	46.6 (8.9, 54.9)	27.6 (8.0, 42.7)
HOMA-IR^a^	2.4 (1.7, 3.8)	3.6 (2.3, 6.8)	2.1 (1.7, 3.1)##
Whole body glucose uptake, mg·kg^−1^·min^−1^	2.5 (1.9, 3.8)	1.9 (1.3, 3.6)	2.8 (2.1, 4.3)
Fasting triglycerides, mmol·L^−1^	1.2 (0.9, 1.7)	1.3 (1.1, 1.7)	1.2 (0.8, 1.5)
Fasting total cholesterol, mmol·L^−1^	4.7 (4.1, 5.2)	4.3 (4.1, 4.7)	4.8 (4.5, 5.4)#
Fasting LDL cholesterol, mmol·L^−1^	3.0 (2.6, 3.5)	2.9 (2.6, 3.2)	3.1 (2.7, 3.8)
Fasting HDL cholesterol, mmol·L^−1^	1.4 (0.3)	1.2 (0.3)	1.5 (0.3)##
Triglycerides:HDL	0.9 (0.6, 1.4)	1.2 (0.6, 1.6)	0.8 (0.6, 1.1)

Values are means (SD) or medians with lower and upper quartiles (Q1, Q3) for nonnormally distributed outcomes. HEC, hyperinsulinemic-euglycemic clamp; V̇o_2_, oxygen uptake; V̇co_2_, carbon dioxide release; RER, respiratory exchange ratio; EE, energy expenditure; CHO, carbohydrate; HOMA-IR, homeostatic model assessment of insulin resistance. ***P < 0.001 between fasting and insulin stimulation in total sample. #*P* < 0.05, ##*P* < 0.01, ###*P* < 0.001 between sexes. ^a^Data available for 63 participants. ^b^Data available for 62 participants.

**Figure 1.**
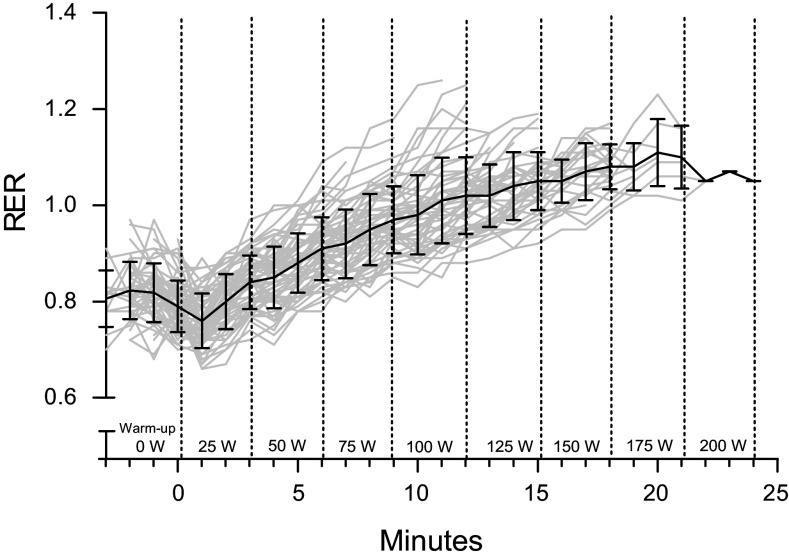
Respiratory exchange ratio (RER) at incrementally increasing exercise loads during a maximal cycle ergometer test. Gray lines represent individual participants (*n* = 64) and black line with error bars indicates the mean (SD).

The mean RER at low-intensity exercise (25 W) was 0.74 (SD 0.05) and at maximal intensity (mean 135 W) 1.12 (SD 0.06) (Table 3), and the increase was statistically significant (*P* < 0.001). The incremental increase in RER with increasing exercise loads is illustrated in Fig. 1. At low-intensity exercise fat was the primary substrate [2.4 (SD 1.0) mg·kg^−1^·min^−1^ vs. CHO 2.2 (1.5) mg·kg^−1^·min^−1^]. At maximal intensity, FATox was entirely suppressed and CHOox increased to 39.2 (SD 8.9) mg·kg^−1^·min^−1^ (*P* < 0.05 for both) (Table 3).

**Table 3.** Metabolic characteristics of the participants during exercise

	Total	Men	Women
Low-intensity exercise^a^			
Power output, W	25	25	25
V̇o_2_, mL·min^−1^	601 (140)	640 (156)	572 (120)
V̇co_2_, mL·min^−1^	471 (110)	516 (119)	437 (90)**
RER	0.74 (0.05)	0.75 (0.05)	0.73 (0.05)
EE, kcal·min^−1^	2.9 (0.7)	3.1 (0.8)	2.8 (0.6)*
CHO oxidation, mg·kg^−1^·min^−1^	2.2 (1.5)	2.9 (1.5)	1.6 (1.3)***
Fat oxidation, mg·kg^−1^·min^−1^	2.4 (1.0)	2.0 (0.8)	2.7 (1.0)**
Maximal exercise^b^			
Max power output, W	135 (103, 151)	150 (147, 165)	112 (98, 135)**
V̇o_2,_ mL·min^−1^	2,127 (482)	2,507 (381)	1,858 (348)***
V̇co_2_, mL·min^−1^	2,345 (524)	2,742 (423)	2,065 (392)***
RER	1.12 (0.06)	1.11 (0.05)	1.13 (0.07)
EE, kcal·min^−1^	10.8 (2.4)	12.7 (1.9)	9.4 (1.8)***
CHO oxidation, mg·kg^−1^·min^−1^	39.2 (8.9)	42.2 (9.6)	37.1 (8.0)*
Fat oxidation, mg·kg^−1^·min^−1^	−4.2 (2.2)	−4.3 (2.1)	−4.2 (2.3)
ΔRER (maximal – low-intensity exercise)	0.39 (0.07)	0.36 (0.06)	0.40 (0.07)*
Delta exercise efficiency, %	16.7 (2.5)	17.4 (1.9)	16.3 (2.8)

Values are means (SD) or medians with lower and upper quartiles (Q1, Q3) for nonnormally distributed outcomes. V̇o_2_, oxygen uptake; V̇co_2_, carbon dioxide release; RER, respiratory exchange ratio; EE, energy expenditure; CHO, carbohydrate. *P < 0.05, **P < 0.01, ***P < 0.001 between sexes. ^a^Data available for 63 participants. ^b^Data available for 58 participants.

### Sedentary Time and PA

**Figure 2.**
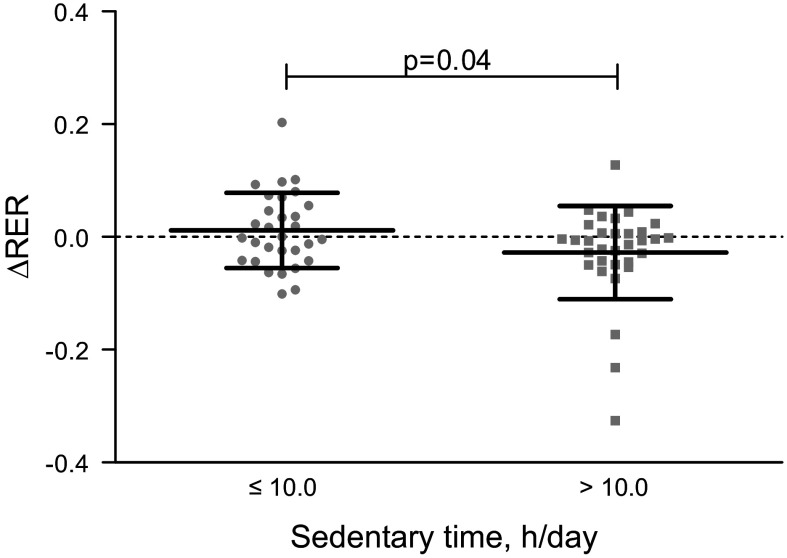
Metabolic flexibility (ΔRER) is better with sedentary time ≤10.0 h/day vs. >10.0 h/day (both *n* = 32). Symbols represent individual participants and black lines with error bars indicate means (SD).

Sedentary time associated positively with fasting RER and inversely with MetFlex (ΔRER) from fasting to insulin stimulation (Table 4). Additionally, MetFlex was better with sedentary time ≤10.0 h/day compared to >10.0 h/day [ΔRER +0.01 (−0.02, 0.04) vs. −0.03 (−0.05, 0.00), respectively, *P* = 0.04] (Fig. 2). Standing time associated negatively with fasting RER, and both LPA and total PA associated positively with insulin-stimulated MetFlex (Table 4).

**Table 4.** Associations of sedentary time, physical activity, and cardiorespiratory fitness with metabolic flexibility from fasting to insulin stimulation

	Fasting RER^a^		Insulin-Stimulated RER		Δ RER Insulin Stimulation^a^	
	β	*P*	β	*P*	β	*P*
Sedentary time, h/day	0.35 (0.04, 0.67)	0.03*	−0.09 (−0.40, 0.23)	0.59	−0.41 (−0.72, −0.09)	0.01*
Standing, h/day	−0.32 (−0.62, −0.02)	0.04*	−0.04 (−0.34, 0.25)	0.77	0.21 (−0.10, 0.52)	0.18
LPA, h/day	−0.09 (−0.38, 0.20)	0.53	0.15 (−0.14, 0.43)	0.31	0. 33 (0.05, 0.61)	0.02*
MVPA, h/day	−0.21 (−0.48, 0.06)	0.13	0.09 (−0.19, 0.36)	0.53	0.18 (−0.09, 0.46)	0.19
Total PA, h/day	−0.17 (−0.45, 0.11)	0.23	0.15 (−0.13, 0.42)	0.30	0.33 (0.05, 0.60)	0.02*
Steps/day	−0.24 (−0.50, 0.02)	0.07	0.09 (−0.18, 0.36)	0.51	0.20 (−0.06, 0.47)	0.13
Breaks in sedentary time/day	−0.14 (−0.42, 0.15)	0.34	−0.08 (−0.36, 0.21)	0.59	0.06 (−0.24, 0.35)	0.71
V̇o_2max_,^b^ mL·kg^−1^·min^−1^	0.07 (−0.23, 0.38)	0.63	0.28 (−0.03, 0.59)	0.07	0.05 (−0.27, 0.37)	0.74
V̇o_2max_,^b^ mL·kg_FFM_^−1^·min^−1^	0.08 (−0.22, 0.38)	0.61	0.13 (−0.17, 0.44)	0.38	0.00 (−0.32, 0.31)	0.98

Values expressed as standardized β-coefficients (95% confidence interval). Model was adjusted for sex, age, and accelerometer wear time for accelerometry outcomes. RER, respiratory exchange ratio; LPA, light-intensity physical activity; MVPA, moderate-to-vigorous physical activity; PA, physical activity; V̇o_2max_, maximal oxygen consumption; FFM, fat-free mass. *P < 0.05, statistical significance. ^a^Data available for 62 participants. ^b^Data available for 58 participants.

**Figure 3.**
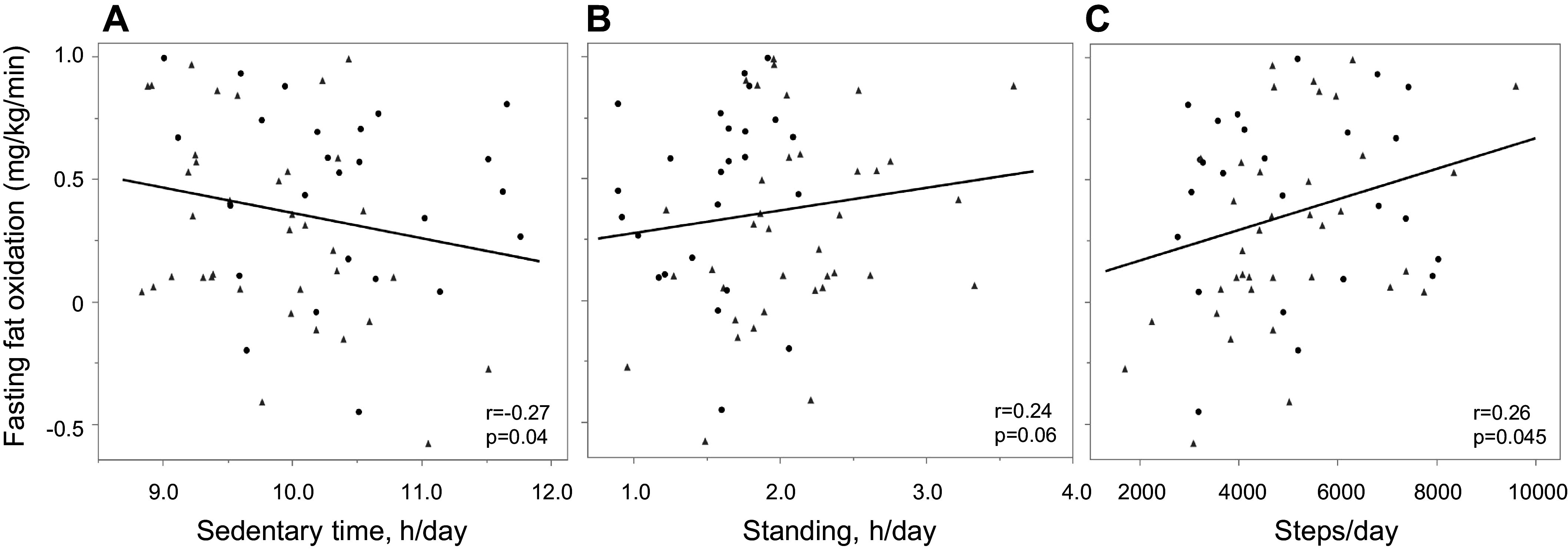
Scatterplots between fasting fat oxidation and sedentary time (*A*), standing time (*B*), and daily steps (*C*). Correlation coefficients are adjusted for sex; solid black circles represent men, and gray triangles women.

Sedentary time also associated with lower fasting FATox, while higher standing time and number of daily steps associated with higher fasting FATox (Table 5; scatterplots illustrated in Fig. 3).

**Table 5.** Associations of sedentary time, physical activity, and cardiorespiratory fitness with fasting and insulin-stimulated substrate oxidation

	Fasting CHOox,^a^ mg·kg^−1^·min^−1^		Fasting FATox,^a^ mg·kg^−1^·min^−1^		Insulin-Stimulated CHOox, mg·kg^−1^·min^−1^		Insulin-Stimulated FATox, mg·kg^−1^·min^−1^	
	β	*P*	β	*P*	β	*P*	β	*P*
Sedentary time, h/day	0.24 (−0.08, 0.57)	0.14	−0.36 (−0.67, −0.04)	0.03*	−0.21 (−0.52, 0.10)	0.19	0.04 (−0.28, 0.35)	0.81
Standing, h/day	−0.29 (−0.59, 0.02)	0.06	0.31 (0.01, 0.61)	0.04*	0.04 (−0.26, 0.33)	0.82	0.06 (−0.24, 0.35)	0.71
LPA, h/day	−0.02 (−0.31, 0.28)	0.90	0.10 (−0.19, 0.39)	0.49	0.18 (−0.10, 0.46)	0.20	−0.11 (−0.39, 0.17)	0.45
MVPA, h/day	−0.10 (−0.38, 0.18)	0.50	0.22 (−0.05, 0.49)	0.11	0.21 (−0.06, 0.48)	0.13	−0.04 (−0.31, 0.24)	0.80
Total PA, h/day	−0.06 (−0.35, 0.23)	0.67	0.18 (−0.10, 0.47)	0.20	0.24 (−0.04, 0.51)	0.09	−0.09 (−0.37, 0.19)	0.51
Steps/day	−0.12 (−0.39, 0.16)	0.39	0.26 (0.00, 0.53)	0.047*	0.22 (−0.04, 0.49)	0.10	−0.03 (−0.29, 0.24)	0.85
Breaks in sedentary time/day	−0.09 (−0.38, 0.20)	0.55	0.17 (−0.12, 0.45)	0.24	0.02 (−0.26, 0.30)	0.88	0.11 (−0.17, 0.39)	0.42
V̇o_2max_,^b^ mL·kg^−1^·min^−1^	0.19 (−0.12, 0.50)	0.22	−0.02 (−0.33, 0.28)	0.88	0.43 (0.13, 0.73)	0.01*	−0.24 (−0.55, 0.07)	0.12
V̇o_2max_,^b^ mL·kg_FFM_^−1^·min^−1^	0.08 (−0.23, 0.38)	0.62	−0.04 (−0.34, 0.26)	0.79	0.16 (−0.14, 0.47)	0.29	−0.13 (−0.43, 0.18)	0.41

Values expressed as standardized β-coefficients (95% confidence interval). Model was adjusted for sex, age, and accelerometer wear time for accelerometry outcomes. CHOox, carbohydrate oxidation; FATox, fat oxidation LPA, light-intensity physical activity; MVPA, moderate-to-vigorous physical activity; PA, physical activity; V̇o_2max_, maximal oxygen consumption; FFM, fat-free mass. *P < 0.05, statistical significance. ^a^Data available for 62 participants. ^b^Data available for 58 participants.

When adjusted for total PA, the associations of sedentary time and standing with fasting RER remained statistically significant (*P* < 0.05). The association of sedentary time with ΔRER, as well as the associations of sedentary time, standing, and steps with fasting FATox, turned nonsignificant (data not shown).

### Exercise and Cardiorespiratory Fitness

Sedentary time nor PA associated with MetFlex during exercise, but higher cardiorespiratory fitness (V̇o_2max_; mL·kg^−1^·min^−1^) associated with higher CHOox during HEC (Table 5), lower RER at low-intensity exercise (Table 6), and higher CHOox during maximal exercise (Table 7). When V̇o_2max_ was expressed per FFM, however, the associations with insulin-stimulated CHOox and low-intensity RER turned nonsignificant. Sedentary time, LPA, MVPA, total PA, and steps all associated with CHOox at maximal exercise intensity, and sedentary time, MVPA, total PA additionally with FATox at low-intensity exercise (Table 7). These associations were mediated through their effects on cardiorespiratory fitness, however, as adjustment for V̇o_2max_ turned all associations nonsignificant (data not shown).

**Table 6.** Associations of sedentary time, physical activity, and cardiorespiratory fitness with metabolic flexibility from low-intensity to maximal exercise

	Low-Intensity Exercise RER^a^		Maximal Exercise RER^b^		ΔRER Exercise^b^	
	β	*P*	β	*P*	β	*P*
Sedentary time, h/day	0.11 (−0.20, 0.42)	0.47	−0.01 (−0.34, 0.32)	0.94	−0.07 (−0.39, 0.24)	0.64
Standing, h/day	0.02 (−0.28, 0.31)	0.92	−0.08 (−0.40, 0.24)	0.61	−0.06 (−0.37, 0.25)	0.71
LPA, h/day	−0.06 (−0.35, 0.23)	0.67	0.20 (−0.11, 0.51)	0.20	0. 17 (−0.13, 0.47)	0.27
MVPA, h/day	−0.22 (−0.50, 0.05)	0.11	−0.09 (−0.38, 0.21)	0.57	0.07 (−0.22, 0.36)	0.64
Total PA, h/day	−0.16 (−0.44, 0.12)	0.26	0.09 (−0.22, 0.39)	0.57	0.15 (−0.14, 0.44)	0.31
Steps/day	−0.20 (−0.47, 0.07)	0.14	−0.11 (−0.40, 0.18)	0.44	0.03 (−0.25, 0.32)	0.75
Breaks in sedentary time/day	0.06 (−0.23, 0.34)	0.70	0.04 (−0.26, 0.35)	0.79	−0.03 (−0.33, 0.27)	0.85
V̇o_2max_,^b^mL·kg^−1^·min^−1^	−0.34 (−0.65, −0.04)	0.03*	−0.05 (−0.37, 0.27)	0.75	0.19 (−0.12, 0.49)	0.23
V̇o_2max_,^b^ mL·kg_FFM_^−1^·min^−1^	−0.24 (−0.54, 0.06)	0.11	0.10 (−0.20, 0.41)	0.50	0.25 (−0.04, 0.54)	0.09

Values expressed as standardized β-coefficients (95% confidence interval). Model was adjusted for sex, age, and accelerometer wear time for accelerometry outcomes. RER, respiratory exchange ratio; LPA, light-intensity physical activity; MVPA, moderate-to-vigorous physical activity; PA, physical activity; V̇o_2max_, maximal oxygen consumption; FFM, fat-free mass. *P < 0.05, statistical significance. ^a^Data available for 63 participants. ^b^Data available for 58 participants.

**Table 7.** Associations of sedentary time, physical activity, and cardiorespiratory fitness with substrate oxidation during exercise

	Low-Intensity Exercise CHOox,^a^ mg·kg^−1^·min^−1^		Low-Intensity Exercise FATox,^a^ mg·kg^−1^·min^−1^		Maximal Exercise CHOox,^b^ mg·kg^−1^·min^−1^		Maximal Exercise FATox,^b^ mg·kg^−1^·min^−1^	
	β	*P*	β	*P*	β	*P*	β	*P*
Sedentary time, h/day	0.09 (−0.19, 0.38)	0.52	−0.32 (−0.61, −0.03)	0.03*	−0.31 (−0.60, −0.03)	0.03*	0.12 (−0.21, 0.44)	0.48
Standing, h/day	0.02 (−0.26, 0.30)	0.88	0.11 (−0.18, 0.40)	0.44	0.07 (−0.22, 0.36)	0.64	0.09 (−0.23, 0.41)	0.59
LPA, h/day	−0.13 (−0.40, 0.14)	0.33	0.26 (−0.01, 0.53)	0.06	0.31 (0.03, 0.58)	0.03*	−0.29 (−0.59, 0.02)	0.06
MVPA, h/day	−0.10 (−0.36, 0.16)	0.46	0.27 (0.01, 0.53)	0.04*	0.29 (0.03, 0.55)	0.03*	−0.07 (−0.37, 0.23)	0.64
Total PA, h/day	−0.14 (−0.40, 0.12)	0.28	0.32 (0.07, 0.58)	0.02*	0.36 (0.10, 0.62)	0.01*	−0.23 (−0.53, 0.07)	0.13
Steps/day	−0.04 (−0.29, 0.21)	0.75	0.22 (−0.03, 0.48)	0.09	0.29 (0.04, 0.55)	0.03*	−0.03 (−0.32, 0.26)	0.83
Breaks in sedentary time/day	0.00 (−0.26, 0.26)	0.99	0.13 (−0.14, 0.40)	0.34	0.23 (−0.04, 0.51)	0.09	−0.13 (−0.44, 0.17)	0.39
V̇o_2max_,^b^ mL·kg^−1^·min^−1^	−0.09 (−0.38, 0.19)	0.51	0.22 (−0.07, 0.52)	0.13	0.81 (0.62, 1.00)	<0.001*	−0.27 (−0.59, 0.04)	0.08
V̇o_2max_,^b^ mL·kg_FFM_^−1^·min^−1^	−0.08 (−0.35, 0.20)	0.58	0.05 (−0.24, 0.33)	0.73	0.69 (0.48, 0.90)	<0.001*	−0.35 (−0.64, −0.05)	0.02*

Values expressed as standardized β-coefficients (95 % confidence interval). Model was adjusted for sex, age, and accelerometer wear time for accelerometry outcomes. CHOox, carbohydrate oxidation; FATox, fat oxidation LPA, light-intensity physical activity; MVPA, moderate-to-vigorous physical activity; PA, physical activity; V̇o_2max_, maximal oxygen consumption; FFM, fat-free mass. *P < 0.05, statistical significance. ^a^Data available for 63 participants. ^b^Data available for 58 participants.

The mean exercise efficiency at moderate-intensity exercise was 16.7 (2.5) %. Higher efficiency correlated with better insulin-stimulated MetFlex, although marginally nonsignificantly (*r* = 0.26, *P* = 0.051). Efficiency was also higher with lower sedentary time, age, body weight, and fat mass (*r* = −0.27, *r* = −0.31, *r* = −0.33, and *r* = −0.30, respectively; *P* < 0.05 for all).

### Glucose, Insulin, and Lactate

Fasting insulin and HOMA-IR correlated inversely with insulin-stimulated CHOox (*r* = −0.26 for both). Insulin also correlated inversely with FATox at low-intensity exercise (*r* = −0.28), and whole body glucose uptake positively with CHOox at maximal exercise intensity (*r* = 0.33) (*P* < 0.05 for all; Supplemental Tables S1–S3 in Supplemental File S1). Higher whole body glucose uptake also correlated with a greater insulin-stimulated increase in lactate and free fatty acid suppression during HEC (*r* = 0.51, and *r* = 0.53, respectively; *P* < 0.001 for both).

Higher fasting lactate correlated with lower insulin-stimulated MetFlex (*r* = −0.35) and fasting FATox (*r* = −0.36), and with higher fasting RER (*r* = 0.36) and CHOox (*r* = 0.43) (*P* < 0.05 for all; Supplemental Tables S1 and S2). Lower fasting lactate and greater insulin-stimulated increase in lactate level both correlated with better free fatty acid suppression (*r* = −0.41 and *r* = 0.35, respectively), as well as lower sedentary time (*r* = 0.35 and *r* = −0.42, respectively) and higher standing time (*r* = −0.40 and *r* = 0.47) (*P* < 0.05 for all).

### Lipids

Fasting triglycerides and triglyceride-HDL ratio both correlated positively with fasting RER (*r* = 0.36 and *r* = 0.28, respectively) and fasting CHOox (*r* = 0.44 and *r* = 0.34) and inversely with fasting FATox (*r* = −0.39 and *r* = −0.32) (*P* < 0.05 for all; Supplemental Tables S1 and S2). Triglycerides also had a positive correlation with insulin-stimulated CHOox (*r* = 0.25). Lower fasting triglycerides and triglyceride-to-HDL ratio correlated with a greater insulin-stimulated increase in lactate (*r* = −0.36 and *r* = −0.44, respectively), and free fatty acid suppression (*r* = −0.33 and *r* = −0.31) (*P* < 0.05 for all). Better fatty acid suppression during HEC also correlated with higher standing time (*r* = 0.28, *P* = 0.03).

### Anthropometrics and Body Composition

CHOox during both insulin stimulation and maximal exercise correlated inversely with weight (*r* = −0.31 and *r* = −0.53, respectively), BMI (*r* = −0.30, *r* = −0.52), waist circumference (*r* = −0.26, *r* = −0.47), and body fat percent (*r* = −0.28, *r* = −0.37) (*P* < 0.05 for all; Supplemental Table S4). Greater increase in lactate during HEC correlated with lower weight (*r* = −0.30), BMI (*r* = −0.30), and waist circumference (*r* = −0.36). Free fatty acid suppression during HEC also correlated inversely with waist circumference (*r* = −0.28) (*P* < 0.05 for all).

### Diet

Protein intake (%total energy intake/day) correlated positively with insulin-stimulated MetFlex (*r* = 0.30). Total daily energy intake correlated inversely with maximal exercise CHOox (*r* = −0.33) and positively with maximal exercise FATox (*r* = 0.33). CHO intake (%total energy intake/day) correlated inversely with FATox at both low- and maximal intensity exercise (*r* = −0.27 and *r* = −0.31, respectively), and positively with CHOox during maximal exercise (*r* = 0.36) (*P* < 0.05 for all; Supplemental Table S5).

### Associations between MetFlex Variables

Higher RER already at a fasting state correlated with lower insulin-stimulated MetFlex (*r* = −0.42), while a higher RER during HEC correlated with better MetFlex in response to insulin stimulation (*r* = 0.45) (*P* ≤ 0.001 for both; Supplemental Table S6). Correspondingly, better insulin-stimulated MetFlex correlated with higher FATox and lower CHOox in a fasting state (*r* = 0.40 and *r* = −0.33, respectively), and higher CHOox and lower FATox during HEC (*r* = 0.46 and *r* = −0.40). Better insulin-stimulated MetFlex also correlated with higher FATox and lower CHOox during low-intensity exercise (*r* = 0.29 and *r* = −0.34) (*P* < 0.05 for all).

Lower RER at low-intensity exercise and higher RER at maximal exercise intensity both correlated with better MetFlex during exercise (*r* = −0.41 and *r* = 0.74, respectively; *P* ≤ 0.001 for both) (Supplemental Table S6). Better MetFlex during exercise correlated with higher CHOox and lower FATox at maximal exercise intensity (*r* = 0.51 and *r* = −0.68, *P* ≤ 0.001 for both).

## DISCUSSION

The main finding of this study is that both lower sedentary time and a higher amount of light-intensity and total PA associate with better MetFlex. To our knowledge, this is the first study to show these associations when PA is assessed with accelerometers and MetFlex with HEC.

### Sedentary Time, PA, and MetFlex

Studies directly investigating the link between PA behaviors and MetFlex are limited. Studies using bed rest models and alternative MetFlex assessment methods (i.e., an index of postprandial RQ and insulin variability) have proposed inactivity and sedentary behavior as primary determinants of MetFlex, suggesting that a high level of PA predicts more flexibility, while inactivity and sedentary behavior trigger a state of inflexibility (5, 10, 11). Our study assessing sedentary time and PA with accelerometers in free-living conditions and MetFlex with HEC now builds on these experimental findings and supports the conclusions, as we found that sedentary time associates with metabolic inflexibility, and more standing, LPA, and total PA associate with more flexibility. Furthermore, sedentary time associated inversely, and standing and steps positively, with fasting FATox, but not CHOox, suggesting that (in)activity may influence MetFlex mainly through effects on lipid metabolism.

A few previous studies have investigated the associations of accelerometer-derived PA and sedentary outcomes with varying definitions of MetFlex. In line with our results, prolonged sedentary time was associated with lower 23-h FATox in healthy young men (22), and an association between sedentary time and FATox was also observed in healthy young women (23). Surprisingly, however, the latter study indicated increased fasting FATox with higher daily sedentary time. Although MetFlex was not directly assessed, similar to our findings the same study also suggested a role for PA in the modulation of energy metabolism by showing an association between MVPA and reduced variance in blood glucose concentration after a glucose-rich meal (23). In our study, however, LPA and total PA, but not MVPA, associated with MetFlex, suggesting that the total amount, bout length, and accumulation pattern of PA might be more important than intensity per se.

Along the same lines, a few experimental studies have indicated that sedentary patterns may impact fuel utilization. Although we did not find an association between MetFlex and breaks in sedentary time, some studies have suggested that more frequent breaks, either by LPA (24) or standing (25), increase FATox. In contrast, another study indicated greater reliance on CHO as fuel with frequent moderate-intensity walking breaks, while an energy-matched single 45-min walking bout increased FATox (26). It may be that in our study the frequency of breaks and/or the intensity of activities breaking up prolonged sitting, for example, were not enough to result in significant associations. However, this can only be speculated as we only assessed the total number of daily sit-to-stand transitions, not the pattern of breaks or activities replacing sitting.

Overall, the findings from this and previous studies suggest that physical (in)activity has an effect on the regulation of fuel metabolism, which is likely modulated through the volume and frequency of muscle contractions, However, the evidence is still scarce and inconclusive and comparisons are difficult to make as the definitions and methods used to assess MetFlex, as well as PA and sedentary behavior, vary between studies. Our study provides novel findings to add to the emerging evidence base and extends the findings from previous studies including mostly healthy participants to a population with existing metabolic impairments.

### Exercise and MetFlex

In impaired glucose tolerance, type 2 diabetes, and metabolic syndrome, the FATox capacity during low-to-moderate exercise is reduced (3, 27, 28). In line with this, instead of reliance on FATox, we observed comparable rates of fatty acid and CHO oxidation at low-intensity exercise, and a progressive increase in RER (reflecting increased CHO use) starting already at the lowest exercise stage and intensity. This is likely due to the accumulation of lactate as a result of reduced lactate clearance capacity (3). Our results also show that better cardiorespiratory fitness is associated with lower RER at a low exercise intensity, indicating a preference for fatty acids over CHO, as well as a better ability to utilize CHO at maximal exercise intensity. Previous studies similarly suggest a link between fitness/training status and MetFlex during exercise (3, 29, 30). Whether fitness is related to MetFlex in response to insulin stimulation, however, is not clear. In our study, fitness did not associate with HEC-measured MetFlex, but CHOox during HEC was higher with better fitness. Additionally, the correlation between exercise efficiency (the ability to transfer consumed energy to mechanical work) and insulin-stimulated MetFlex was nearly significant. A positive association between fitness and HEC-measured MetFlex has previously been reported in healthy young men (31) but not in adults with obesity or newly diagnosed type 2 diabetes (8, 32). Along the same lines, the association between fitness and insulin-stimulated CHOox turned nonsignificant in our study when fitness was expressed per FFM, indicating that body composition is likely a more important determinant of insulin-stimulated substrate oxidation than fitness status. More research is thus needed to establish whether cardiorespiratory fitness plays a role in modulating energy metabolism during physiological conditions other than exercise as well.

### Physiological Considerations

Our results show that fasting FATox is low and the ability to switch between fat and carbohydrate oxidation in response to insulin stimulation is blunted in sedentary and physically inactive adults with metabolic syndrome. This is in agreement with a large body of evidence indicating metabolic inflexibility in response to different metabolic and physiological challenges (e.g., insulin stimulation, varying dietary compositions, exercise) in obesity, insulin resistance, metabolic syndrome, and type 2 diabetes (2–4, 27, 33–37). The physiological mechanisms explaining impairments in MetFlex are complex and multifactorial, but, e.g., skeletal muscle characteristics (2), glucose disposal rate (33, 35), impaired suppression of adipose tissue lipolysis (38), the capacity for glycolysis and beta-oxidation (2, 39), and mitochondrial dysfunction (35, 40) are suggested to play a role. Differences in energy balance and macronutrient dietary composition, as well as genetics and hereditary factors, may also have an influence on FATox capacity and MetFlex (41, 42). Plasma concentrations of glucose and free fatty acids are also suggested to be key determinants of MetFlex (7, 33, 35). Multiple factors and disturbances in several different steps on the metabolic pathway may thus contribute to the development of metabolic inflexibility. Moreover, it is unclear whether MetFlex responses to different metabolic challenges (e.g., fasting, HEC, exercise) are regulated by similar mechanisms; for example, in our study neither fasting RER nor insulin-stimulated MetFlex correlated with MetFlex during exercise and the associations with activity outcomes were different.

In our study, MetFlex did not associate with the intake of total energy or primary energy substrates CHO and fat. Interestingly, however, protein intake correlated positively with insulin-stimulated MetFlex. The contribution of protein itself to overall fuel oxidation is minor, but it is possible that the effects on insulin and glucagon secretion, and/or the role of amino acids in gluconeogenesis mediate this association (43). We did not find any statistically significant associations between fasting RER or insulin-stimulated MetFlex and fasting glucose or free fatty acid concentrations, insulin-stimulated glucose uptake, nor free fatty acid suppression during HEC either (data not shown). Although not directly assessed, it may be speculated that the metabolic inflexibility observed in our study could potentially be related to reduced mitochondrial capacity. Indeed, in support of this speculation, insulin resistance, type 2 diabetes, and metabolic syndrome are often associated with reduced mitochondrial content and/or function (44). Additionally, we found an association between fasting blood lactate level and metabolic inflexibility, which supports the hypothesis of reduced mitochondrial capacity, and/or dysregulated glycolytic activity. Lactate metabolism is closely tied to mitochondrial function and high levels of lactate limit and downregulate FATox (3, 44), as also indicated by the inverse correlation between fasting lactate and FATox in our study. Both lower fasting lactate and greater insulin-stimulated increase in lactate during HEC also correlated with less sedentary time and more standing time, thus further supporting the role of habitual activity behaviors in the regulation of energy metabolism.

Insulin resistance has consistently been shown to be one of the key components of metabolic inflexibility (2, 8, 32, 33, 36), but in our study MetFlex did not associate statistically significantly with neither HEC-measured whole body glucose uptake nor HOMA-IR, although the latter was only marginally nonsignificant (*P* = 0.054). However, whole body glucose uptake associated with a better ability to suppress lipolysis and increase lactate levels in response to insulin and to oxidize CHO during maximal exercise. Correlations were also found between substrate oxidation variables and surrogate markers of insulin sensitivity (i.e., HOMA-IR, fasting insulin, triglyceride/HDL ratio). Our results thus also indicate a link between MetFlex and insulin sensitivity.

Insulin resistance is tightly connected with impairments in lipid metabolism, which are likely responsible for the reduced MetFlex in our study as well. This is indicated by the low fasting FATox rate and further supported by the associations of plasma triglycerides with MetFlex and substrate oxidation variables. Higher circulating triglycerides correlated positively with higher CHOox and lower FATox in a fasting state, which suggests increased lipogenesis and triglyceride synthesis from oxidized glucose. Impaired muscle lipoprotein lipase activity could also have contributed to the associations between triglycerides and substrate oxidation by reducing the hydrolysis of triglycerides and consequently the amount of free fatty acids available for oxidation (45).

### Strengths and Limitations

A few studies have assessed sedentary time, PA, and MetFlex with varying, often surrogate measures, and to our knowledge, we now show for the first time that accelerometer-assessed sedentary time and PA associate with MetFlex assessed with HEC. Consequently, the 4-wk accelerometer measurement and HEC can be considered the key strengths of our study. It is worth mentioning, however, that insulin stimulation by HEC is not a physiological challenge; therefore, assessing MetFlex during exercise as well is a strength. Limitations include the cross-sectional setting and the relatively small, homogenous sample of sedentary inactive adults with metabolic syndrome. Another potentially confounding factor might be considered the timing of the second calorimetry measurement, as it was started relatively shortly after the initiation of HEC, which may have influenced the results. However, low FATox already in the first measurement after an overnight fast is an implication of impaired MetFlex, and the mean blood glucose concentration of 5.0 mmol/l and CV of 5.5% (SE 0.6) during the 30- to 60-min period of HEC when the second calorimetry measurement was performed suggests that steady-state was achieved in HEC (Supplemental Fig. S1 in Supplemental File S1). Additionally, the preceding diet and the time of day for the fitness test were not controlled for, nor was the diet in the preceding days before HEC. However, the participants were instructed to fast overnight and avoid strenuous physical exertion, caffeine, and alcohol for 24 h before research visits, and none of the MetFlex outcomes associated with the intake of total energy or primary energy substrates CHO and fats reported with the food diaries, which, according to the given instructions, should reflect the participants’ usual dietary habits. Furthermore, the effect of potential nutritional differences is minimized by controlling the glucose and insulin concentrations during HEC.

### Conclusions

This study shows that high sedentary time associates with impaired fasting lipid metabolism and metabolic inflexibility, while more standing and PA associate beneficially with fasting FATox and MetFlex in sedentary and physically inactive adults with metabolic syndrome. Although causality cannot be determined due to the cross-sectional study setting, the findings suggest that reducing sedentary time and increasing PA of even light intensity may help in the prevention of metabolic diseases in risk populations through their potential effects on fuel utilization, particularly lipid metabolism.

## DATA AVAILABILITY

Data are available from the corresponding author on a reasonable request.

## SUPPLEMENTAL MATERIAL

10.5281/zenodo.10567014 Supplemental Fig. S1 and Supplemental Tables S1–S6 (included in Supplemental File S1): https://doi.org/10.5281/zenodo.10567014.

## GRANTS

This work was supported by the Research Council of Finland, Finnish Cultural Foundation, Finnish Diabetes Research Foundation, Hospital District of Southwest Finland, and Juho Vainio Foundation.

## DISCLOSURES

Outside of this work, J.K. has received consultancy fees from GE Healthcare and AstraZeneca and speaker fees from GE Healthcare, Bayer, Lundbeck, Boehringer-Ingelheim, and Merck; and T.S. has received a speaker fee from Pihlajalinna. The other authors declare that they have nothing to disclose.

## AUTHOR CONTRIBUTIONS

T.S., K.K.K., T.V., J.K., and I.H. conceived and designed research; T.G., T.S., S.L., M.K., P.K., M.S., N.H., and K.L. performed experiments; T.G., T.S., S.L., H.V.-Y, T.V., J.N., E.L., H.S., and I.H., analyzed data; T.G., T.S., S.L., H.V.-Y, T.V., J.N. E.L., H.S., and I.H. interpreted results of experiments; T.G. prepared figures; T.G. drafted manuscript; T.G., T.S., S.L., M.K., H.V.-Y, T.V., J.N., P.K., M.S.. E.L., H.S., N.H., K.L., K.K.K., T.V., J.K., and I.H. edited and revised manuscript; T.G., T.S., S.L., M.K., H.V.-Y, T.V., J.N., P.K., M.S., E.L., H.S., N.H., K.L., K.K.K., T.V., J.K., and I.H. approved final version of manuscript.
